# Supplementary material for: A Systematic Review and Meta-Analysis of the Clinical Use of Megestrol Acetate for Cancer-Related Anorexia/Cachexia
Source: J Clin Med. 2022 Jun 28;11(13):3756. doi: 10.3390/jcm11133756 (PMC9267332; doi:10.3390/jcm11133756)
Supplement: Supplementary file 1 [file jcm-11-03756-s001.zip › jcm-1768826-supplementary.pdf]

Supplementary Material

Table S1. Risk of bias assessment of included studies

	Random sequence generation (selection bias)	Allocation concealment (selection bias)	Blinding of participants and personnel (performance bias)	Blinding of outcome assessment (detection bias)	Incomplete outcome data (attrition bias)	Selective reporting (reporting bias)	Other bias	Overall
Abrams et al, 1999	Green	Green	Yellow	Yellow	Green	Green	Yellow	Yellow
Beller et al, 1997	Green	Green	Green	Green	Red	Green	Yellow	Red
Chao et al, 1997	Red	Red	Red	Red	Red	Green	Green	Red
Chow et al, 2011	Green	Green	Green	Yellow	Red	Green	Green	Red
Collichio et al, 1998	Red	Red	Red	Red	Red	Green	Yellow	Red
Couluris et al, 2008	Red	Red	Red	Red	Green	Green	Yellow	Red
Currow et al, 2021	Green	Green	Green	Green	Red	Green	Green	Red
Cuvelier et al 2014	Green	Green	Green	Yellow	Green	Green	Yellow	Yellow
Greig et al 2014	Red	Red	Red	Red	Red	Green	Green	Red
Guo et al, 2002	Green	Red	Red	Red	Green	Green	Yellow	Red
Jatoi et al, 2002	Green	Green	Green	Yellow	Red	Green	Yellow	Red
Jatoi et al, 2004	Green	Green	Green	Yellow	Red	Green	Green	Red
Levitan et al, 1998	Red	Red	Red	Red	Green	Green	Red	Red
Loprinzi et al, 1999	Green	Yellow	Red	Red	Red	Green	Yellow	Red
Maddedu et al, 2012	Green	Yellow	Yellow	Yellow	Red	Green	Green	Red
Mantovani et al, 2008	Green	Yellow	Yellow	Yellow	Green	Green	Yellow	Yellow
McMillan et al, 1994	Green	Green	Green	Green	Red	Green	Yellow	Red
McMillan et al, 1999	Green	Yellow	Yellow	Yellow	Red	Green	Green	Red
Navari et al, 2010	Green	Yellow	Red	Red	Green	Green	Yellow	Red

Nelson et al, 2002
Rowland Jr et al, 1996
Tanca et al, 2009
Wen et al, 2012

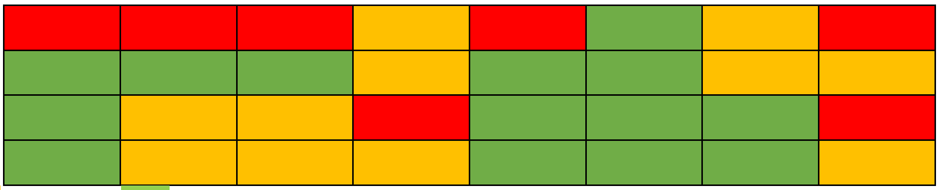

Abbreviation: high, unclear and low risk of bias
